# Piperine Alleviates Doxorubicin-Induced Cardiotoxicity via Activating PPAR-*γ* in Mice

**DOI:** 10.1155/2019/2601408

**Published:** 2019-12-17

**Authors:** Jie Yan, Si-Chi Xu, Chun-Yan Kong, Xiao-Yang Zhou, Zhou-Yan Bian, Ling Yan, Qi-Zhu Tang

**Affiliations:** ^1^Department of Cardiology, Renmin Hospital of Wuhan University, Wuhan 430060, China; ^2^Hubei Key Laboratory of Metabolic and Chronic Diseases, Wuhan 430060, China

## Abstract

**Background:**

Oxidative stress, inflammation and cardiac apoptosis were closely involved in doxorubicin (DOX)-induced cardiac injury. Piperine has been reported to suppress inflammatory response and pyroptosis in macrophages. However, whether piperine could protect the mice against DOX-related cardiac injury remain unclear. This study aimed to investigate whether piperine inhibited DOX-related cardiac injury in mice.

**Methods:**

To induce DOX-related acute cardiac injury, mice in DOX group were intraperitoneally injected with a single dose of DOX (15 mg/kg). To investigate the protective effects of piperine, mice were orally treated for 3 weeks with piperine (50 mg/kg, 18:00 every day) beginning two weeks before DOX injection.

**Results:**

Piperine treatment significantly alleviated DOX-induced cardiac injury, and improved cardiac function. Piperine also reduced myocardial oxidative stress, inflammation and apoptosis in mice with DOX injection. Piperine also improved cell viability, and reduced oxidative damage and inflammatory factors in cardiomyocytes. We also found that piperine activated peroxisome proliferator-activated receptor-*γ* (PPAR-*γ*), and the protective effects of piperine were abolished by the treatment of the PPAR-*γ* antagonist in vivo and in vitro.

**Conclusions:**

Piperine could suppress DOX-related cardiac injury via activation of PPAR-*γ* in mice.

## 1. Introduction

A medical survey conducted by National Health and Nutrition Examination, which included 1807 cancer survivors, showed that 33% died of heart diseases [1]. As a representative drug of anthracycline, doxorubicin (DOX) is one of the major culprits in chemotherapy-induced cardiotoxicity, which could lead to irreversible degenerative cardiomyopathy and heart failure [2]. Because of the tremendous burden of managing DOX-induced cardiotoxicity, rapid discovery of effective treatments would be of great significance.

The pathogenesis of DOX-induced cardiotoxicity are not completely understood, but increasing evidence suggests that oxidative stress, inflammation accumulation and cardiac apoptosis are closely involved [3, 4]. We previously found suppressing apoptosis prevented DOX-induced cardiomyopathy in mice [5]. It has been reported that activation of peroxisome proliferator-activated receptor-*γ* (PPAR-*γ*) by pioglitazone ameliorated cardiac oxidative stress and inflammation in rats with metabolic syndrome [6]. PPAR-*γ* activation inhibited septic-related cardiac dysfunction via attenuation of apoptosis in rats [7]. Moreover, PPAR-*γ* mRNA and protein expression were significantly decreased in mice with DOX treatment [8], and upregulation of PPAR-*γ* antagonized DOX-induced cardiotoxicity in cardiac cells [9]. The findings highlighted the possibility of developing therapeutic strategies targeting PPAR-*γ* to treat DOX-related cardiac injury.

Piperine is the bioactive alkaloid ingredient of black pepper and long pepper [10]. Piperine presented a diverse of biological activities including immune modulation, anti-depressive disorders and mitigating obesity and diabetes [11]. It is noteworthy that piperine could reduce the production of type I interferon and antagonize lipopolysaccharide-induced inflammatory responses [12]. Piperine also significantly inhibited the release of inflammatory factors and pyroptosis in lipopolysaccharide-primed macrophages by activating AMP-activated protein kinase [13]. The data in our lab demonstrated that piperine was a moderate agonist of PPAR-*γ* and could attenuate pathological cardiac fibrosis in mice [14]. However, whether piperine could protect the mice against DOX-related cardiac injury remain unclear. Here, we have shown that piperine attenuated cardiac injury and improved cardiac function in DOX-treated mice via activation of PPAR-*γ*.

## 2. Materials and Methods

### 2.1. Reagents

Piperine (≥97% purity) was obtained from Shanghai Winherb Medical Science Co. (Shanghai, China). DOX (D1515, purity ≥98%) and GW9662 (M6191, purity ≥98%) were purchased from Sigma-Aldrich (St. Louis, MO, USA). The following first antibodies were purchased from Abcam (Cambridge, UK): anti-nuclear factor κB (NF-κB, ab16502, 1 : 1000), anti-GAPDH (ab181602, 1 : 1000), anti-proliferating cell nuclear antigen (PCNA, ab92552, 1 : 1000), anti-tumor necrosis factor-*α* (TNF-*α*, ab6671, 1 : 1000), anti-nuclear factor erythroid-2-related factor 2 (Nrf2, ab76026, 1 : 1000), anti-Bcl-2 (ab185002, 1 : 1000), cleaved-caspase3 (ab2302, 1 : 1000). The N-terminal pro brain natriuretic peptide (NT-proBNP) detection kit was provided by My BioSource (CA, USA). Cardiac troponin I (cTnI) detection kit was purchased from Life Diagnostics, Inc (West Chester, PA).

### 2.2. Animals and Treatments

All animal experimental procedures were in accordance with the Guidelines for the Animal Use Committees of our hospital and our institute. All the C57BL/6 mice (Age: 8–10 weeks, 24–26 g) were divided into four groups (*n* = 12 each group): normal saline (NS) + vehicle, NS + piperine, DOX + vehicle and DOX + piperine, by a random number table. The dose of piperine was selected according to our previous study [14]. To investigate the protective effects of piperine, mice were orally treated for 3 weeks with piperine (50 mg/kg, 18:00 every day), which was diluted in DMSO (0.1% v/v), beginning two weeks before DOX injection. Mice in the vehicle group were given the same volume of DMSO (0.1% v/v). To induce DOX-related acute cardiac injury, mice in DOX group were intraperitoneally injected with a single dose of DOX (15 mg/kg) and the control animals were subjected to equal volume of NS. One week post DOX injection, invasive hemodynamic monitoring was performed and after that these mice were killed with an overdose of sodium pentobarbital and the hearts were collected for further detection. To verify the hypothesis that piperine exerted its cardioprotection via activating PPAR-γ, mice were treated with a PPAR-γ inhibitor (GW9662, 0.35 mg/kg per day in drinking water) for 2 weeks beginning one week before DOX injection as previously described [15].

### 2.3. HE Staining

The heart were fixed with neutral formalin and then processed by standard histological protocol and stained with haematoxylin and eosin (HE). The sections were observed to find pathological alterations caused by DOX.

### 2.4. Hemodynamics

Invasive hemodynamic monitoring was performed according to our previous studies [14, 16, 17]. Left ventricular performance was analyzed in anesthetized mice (isoflurane 1.5% v/v) by using 1-F microtip pressure-volume catheter, which was connected to a Millar Pressure-Volume System (MPVS-400; Millar Instruments) and the data were analyzed using PVAN data analysis software.

### 2.5. Western Immunoblot

Protein was extracted from the heart samples using RIPA lysis buffer containing protease inhibitor and phosphatase inhibitor cocktail. Proteins were fractionated on SDS–PAGE and then transferred onto polyvinylidene difluoride (PVDF) membrane (Invitrogen) [18, 19]. After incubating with the first antibodies at 4°C  overnight and the second antibodies at room temperature for one hour, these membranes were scanned with enhanced chemiluminescence reagent and visualized using the BIO-RAD ChemiDoc Touch Imaging System (BIO-RAD, Hercules, CA, USA). GAPDH was used as the internal control. The nuclear protein was prepared according to the manufacturer's instructions (Thermo Fisher Scientific, MD, USA) and PCNA was used as internal control.

### 2.6. Quantitative Real-Time PCR

The frozen heart tissues or cell lysates were lysed using TRIzol reagent (Invitrogen Life Technologies, USA) to extract the total RNA. After that, total RNA was reversely transcribed to cDNA using Transcriptor First Strand cDNA Synthesis Kit (Roche, USA). Real-time PCR was performed using LightCycler 480 SYBR Green Master Mix (Roche Diagnostics). The expression levels of the target genes were normalized to that of GAPDH.

### 2.7. Terminal Deoxynucleotidyl Transferase-Meditated dUTP Nick-End Labeling (TUNEL) Staining

Fresh heart samples were cut into sections and fixed in paraformaldehyde (4%, ml/ml) at room temperature. TUNEL detection was performed using ApopTag Peroxidase In Situ Apoptosis Detection Kit (Chemicon, CA, USA). For each animal, five sections were selected to count apoptotic nuclei. For each slide 8 fields were randomly chosen.

### 2.8. Antioxidant Assay, Determination of cTnI, NT-ProBNP, Lactate Dehydrogenase and Caspase3 Activity

The heart tissue was homogenized and the supernatant fraction was collected to detect activities antioxidant enzyme. The levels of malondialdehyde (MDA) and 3-nitrotyrosine (3-NT), and the activity of total superoxide dismutase (SOD) were assayed according to the manufacturer's protocols.

MDA assay kit (No: A003-1-2) and SOD activity assay kit (No: A001-3-2) were obtained from Nanjing Jiancheng Bioengineering Institute (Nanjing, China). 3-NT detection kit was provided by Abcam (No: ab116691).

Three days after DOX injection, blood was collected from the retro-orbital plexus to detect cTnI and NT-pro BNP to reflect cardiac injury using the commercial kits.

After treatment, fresh hearts or cells were homogenized to detect lactate dehydrogenase (LDH) release and the activity of caspase3. The level of LDH and caspase3 activity were assayed using kits from Nanjing Jiancheng Bioengineering Institute according to the manufacturer's protocols.

### 2.9. Cell Culture and Treatment

The H9c2 cells were purchased from the American Type Culture Collection (ATCC, Rockville, MD, USA). Cells were routinely seeded in DMEM (Gibco, NY, USA) containing 10% fetal bovine serum (Gibco), penicillin (100 U/ml) and streptomycin (100 U/ml) (Gibco). Only cells below passage 10 were used in our study. H9c2 cells were treated with DOX (1 *μ*mol/L) or PBS for 24 h to mimic DOX-related cardiac injury in vivo. Meanwhile, piperine (20 *μ*mol/l) dissolved in DMSO (0.1% v/v) was given [14]. For PPAR-*γ* inhibition, H9c2 cells were given a specific PPAR-*γ* antagonist (GW9662, 10 *μ*mol/l) for 48 h beginning from 24 h before piperine and DOX treatment [14]. To evaluate PPAR-*γ* transactivation after piperine treatment, H9c2 cells were electrotransfected with Ppre-luc (0.04 *μ*g) and Ppar-*γ* plasmid (0.4 *μ*g) using Neon® Transfection System (pulse voltage: 1750 V, pulse width: 25 ms) [14]. H9c2 cells were harvested 48 h later and luciferase assays performed using a Single-Mode SpectraMax® Microplate Reader to detect the transactivation of PPAR-*γ*. Cell viability after piperine treatment was determined using the CCK-8 kit according to the manufacturer's instructions.

### 2.10. Statistical Analysis

All the data are expressed as mean ± SD. Differences were analysed using one-way analysis of variance (ANOVA) and post hoc analysis was performed with the Tukey test in SPSS software package. Statistical significance was accepted at a value of *P* < 0.05.

## 3. Results

### 3.1. Piperine Attenuated Cardiac Injury in Mice with DOX Injection

**Figure 1:**
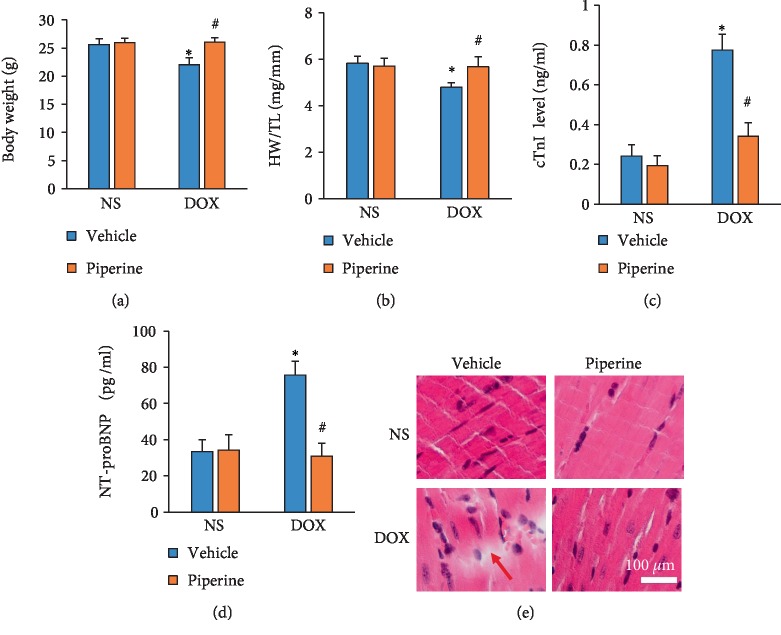
Effects of piperine treatment on DOX-related cardiac injury in mice. (a) Body weight (*n* = 12). (b) The ratio of heart weight to tibia length (*n* = 12). (c) The levels of cTnI in the indicated groups (*n* = 6). (d) The levels of NT-proBNP in the indicated groups (*n* = 6). (e) HE staining. Arrow indicated interruption of myofibrillar. Data are expressed as mean ± SD. ^∗^*P* < 0.05 vs NS/vehicle group; ^#^*P* < 0.05 vs DOX/vehicle group. Data were analyzed sing one-way ANOVA, followed by Tukey post hoc analysis.

The mice subjected to DOX injection had decreased body weight and heart weight/tibia length (HW/TL). However, these pathological alterations in DOX-treated mice were significantly reduced after piperine treatment (Figures 1(a) and 1(b)). Plasma levels of cTnI is a best-known marker of cardiac injury. DOX injection resulted in a marked increase in the level of cTnI, and piperine treatment significantly decreased this elevation in DOX-injected mice (Figure 1(c)). The level of NT-proBNP was increased in DOX group, and piperine treatment decreased the increased level of NT-proBNP (Figure 1(d)). To further evaluate the effect of piperine on DOX-induced cardiac injury, HE staining was performed. Piperine largely reduced interruption of myofibrillar in DOX-treated mice (Figure 1(e)).

### 3.2. Piperine Improved Cardiac Function in Mice Subjected to DOX Injection

**Figure 2:**
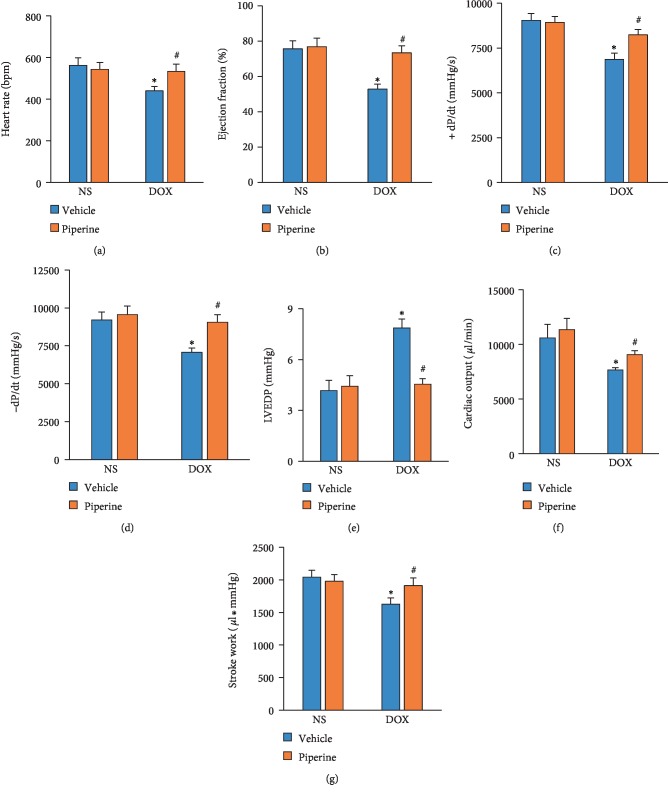
Effects of piperine treatment on hemodynamics in mice. (a) Heart rate (*n* = 8). (b) Ejection fraction (*n* = 8). (c) and (d) The alterations in ±dp/dt in the mice (*n* = 8). (e) LVEDP in the indicated groups (*n* = 8). (f) and (g) Cardiac output and stroke work in the indicated groups (*n* = 8). Data are expressed as mean ± SD. ^∗^*P* < 0.05 vs. NS/vehicle group; ^#^*P* < 0.05 vs. DOX/vehicle group. Data were analyzed sing one-way ANOVA, followed by Tukey post hoc analysis.

DOX injection decreased heart rate in mice, this toxic effect was attenuated after piperine treatment (Figure 2(a)). The mice subjected to DOX injection developed deteriorated cardiac function, as indicated by the decrease in ejection fraction (EF), dP/dt max and dP/dt min (Figures 2(b)–2(d)). Piperine treatment restored impaired cardiac contractility in mice (Figures 2(b)–2(d)). Compared with mice in NS group, mice subjected to DOX injection developed a marked increase of left ventricular end diastolic pressure (LVEDP) and a decrease of cardiac output and stroke work (Figures 2(e)–2(g)). Conversely, compared with mice with DOX injection, these pathological response was attenuated in mice with piperine treatment (Figures 2(e)–2(g)).

### 3.3. Piperine Efficiently Blocked DOX-Induced Inflammation Accumulation in Mice

**Figure 3:**
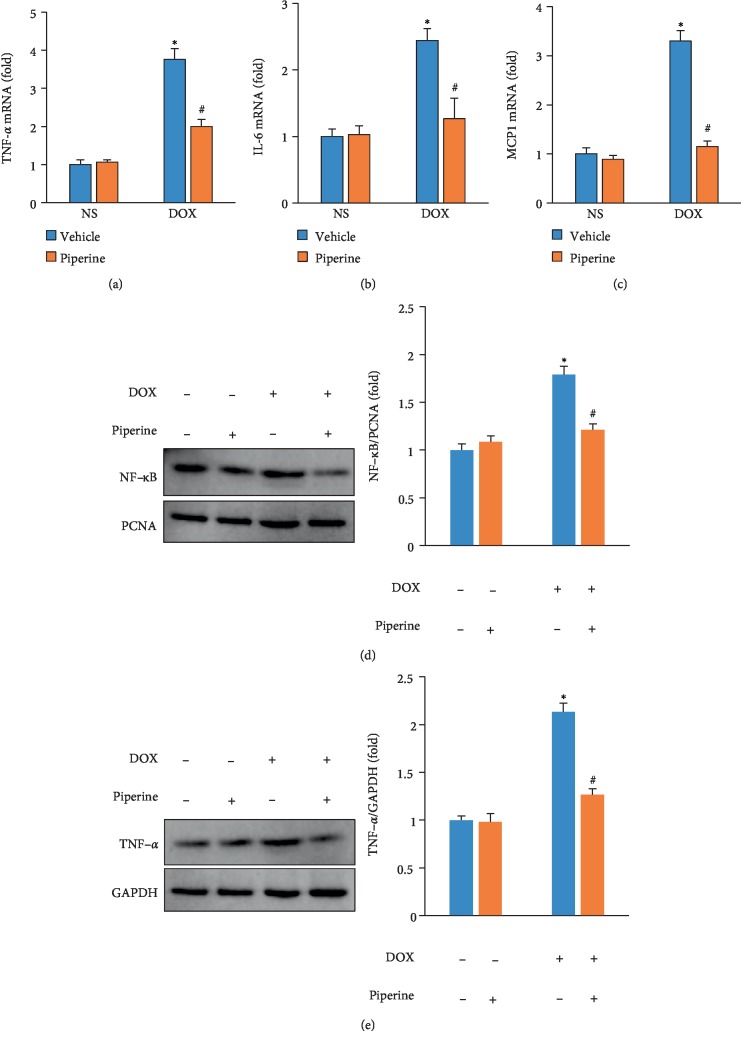
Effects of piperine treatment on inflammatory response in mice. (a)–(c) The levels of inflammatory factors in mice (*n* = 6). (d) The level of nuclear NF-κB in mice (*n* = 6). (e) The blot of TNF-*α* and statistical results (*n* = 6). Data are expressed as mean ± SD. ^∗^*P* < 0.05 vs. NS/vehicle group; ^#^*P* < 0.05 vs. DOX/vehicle group. Data were analyzed sing one-way ANOVA, followed by Tukey post hoc analysis.

Inflammation is one of major features of acute cardiotoxicity caused by DOX injection in mice [20]. Therefore, we detected the effect of piperine on myocardial inflammation in DOX-treated mice. DOX-treated mice had higher levels of TNF-*α*, interleukin (IL)-6, and monocyte chemoattractant protein 1 (MCP-1) compared with mice with NS injection (Figures 3(a)–3(c)). These increased inflammatory factors were attenuated after piperine treatment (Figures 3(a)–3(c)). DOX stimulated nuclear NF-κB accumulation in mice; this effect was inhibited by piperine treatment (Figure 3(d)). Further detection of TNF-*α* protein expression also revealed that piperine reduced cardiac TNF-*α* expression in mice from DOX group (Figure 3(e)).

### 3.4. Piperine Attenuated DOX-Induced Cardiac Oxidative Stress in Mice

**Figure 4:**
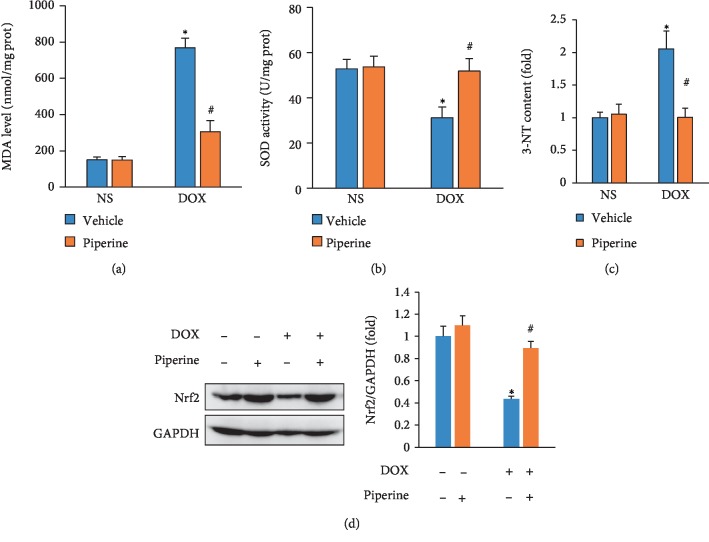
Effects of piperine treatment on oxidative damage in mice. (a) The levels of MDA in mice (*n* = 6). (b) SOD activity in mice (*n* = 6). (c) The level of 3-NT in the indicated groups (*n* = 6). (d) The blot of Nrf2 and statistical results (*n* = 6). Data are expressed as mean ± SD. ^∗^*P* < 0.05 vs. NS/vehicle group; ^#^*P* < 0.05 vs. DOX/vehicle group. Data were analyzed sing one-way ANOVA, followed by Tukey post hoc analysis.

To determine whether piperine could inhibit DOX-induced oxidative damage in mice, we compared the levels of MDA and 3-NT, and the total activity of SOD in mice. Results of these studies showed that piperine could significantly reduce the levels of MDA and 3-NT, and upregulated SOD activity in mice (Figures 4(a)–4(c)). Further detection also revealed that piperine restored Nrf2 expression to the normal level in mice with DOX injection (Figure 4(d)).

### 3.5. Piperine Inhibited Cardiomyocytes Apoptosis in response to DOX

**Figure 5:**
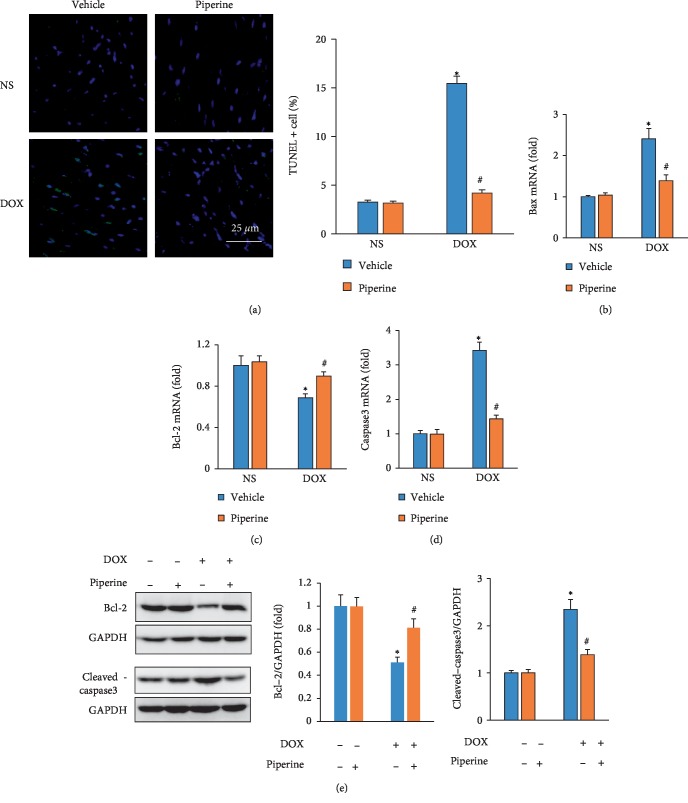
Effects of piperine treatment on apoptosis in mice. (a) TUNEL staining in mice (*n* = 6). (b)–(d) The level of Bax, Bcl-2 and caspase3 in the indicated groups (*n* = 6). (e) The blot of Bcl-2 and cleaved-caspase3 (*n* = 6). Data are expressed as mean ± SD. ^∗^*P* < 0.05 vs. NS/vehicle group; ^#^*P* < 0.05 vs. DOX/vehicle group. Data were analyzed sing one-way ANOVA, followed by Tukey post hoc analysis.

Next, we further evaluated apoptosis using the TUNEL assay. We observed a reduction in the TUNEL + cells in mice from DOX + piperine group compared with that of mice from DOX + vehicle group (Figure 5(a)).Piperine also downregulated the mRNA levels of Bax (pro-apoptotic gene) and caspase3 but upregulated the mRNA level of Bcl-2 (anti-apoptotic gene) in DOX-treated mice (Figures 5(b)–5(d)). Further detection revealed that piperine also upregulated the protein expression of Bcl-2 and decreased cleaved-caspase3 in mice with DOX injection (Figure 5(e)).

### 3.6. Piperine Inhibited DOX-Related Cardiomyocytes Injury In Vitro via Activating PPAR-*γ* Receptor

**Figure 6:**
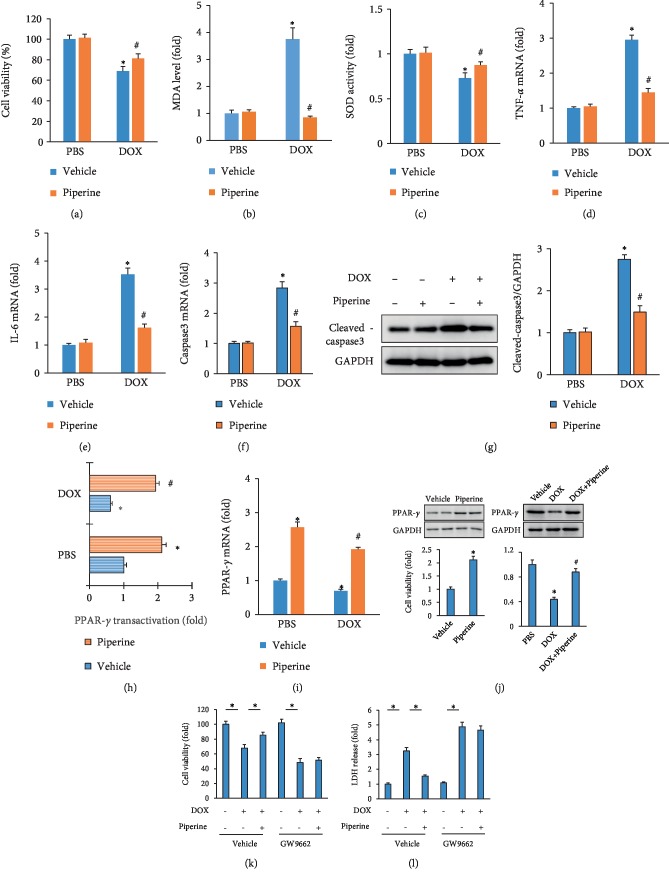
Effects of piperine treatment on oxidative stress, inflammation and apoptosis in cells. (a) Cell viability (*n* = 6). (b) and (c) MDA level and SOD activity (*n* = 6). (d) and (e) The levels of inflammatory factors in mice (*n=6*). (f) and (g) Caspase3 mRNA level and cleaved-caspase3 blot (*n* = 6). (h) The luciferase assay (*n* = 6). (i) The level of PPAR-*γ* (*n* = 6). (j) PPAR-*γ* protein expression (*n* = 6). (k) and (l) Cell viability and LDH release (*n* = 6). Data are expressed as mean ± SD. For (a)–(g) ^∗^*P* < 0.05 vs. PBS/vehicle group; ^#^*P* < 0.05 vs. DOX/vehicle group. For (h) and (i), ^∗^*P* < 0.05. Data were analyzed sing one-way ANOVA, followed by Tukey post hoc analysis.

To further investigate the effect of piperine on DOX-induced damage, H9c2 cardiomyocytes were used. Dox significantly reduced the cell viability of cardiomyocytes, and this effect was attenuated by piperine treatment (Figure 6(a)). Piperine also reduced MDA content and increased SOD activity in DOX-treated cells (Figures 6(b) and 6(c)). The increased mRNA level of TNF-*α* and IL-6 in DOX-treated cells were also suppressed by piperine in vitro (Figures 6(d) and 6(e)). Piperine also reduced caspase3 mRNA and cleaved-caspase3 protein level in DOX-treated cells (Figures 6(f) and 6(g)). Consistent with our study [14], we found that piperine (20 *μ*mol/l) could transactivate PPAR-*γ* and also exhibited a 2.5-fold increase in PPAR-*γ* gene in H9c2 even at baseline (Figure 6(h)). DOX decreased the transactivation and the mRNA expression of PPAR-*γ* in vitro, and piperine treatment largely inhibited this pathological alterations in DOX-treated cells (Figure 6(i)). Piperine increased PPAR-*γ* protein expression at baseline, and also restored PPAR-*γ* protein expression in DOX-treated cells (Figure 6(j)). GW9662 pretreatment, an irreversible antagonist of PPAR-*γ*, completely blocked the protective effects of piperine on cell viability and LDH release in vitro (Figures 6(k) and 6(l)).

### 3.7. Piperine Lost Cardiac Protection in Mice with GW9662 Treatment

**Figure 7:**
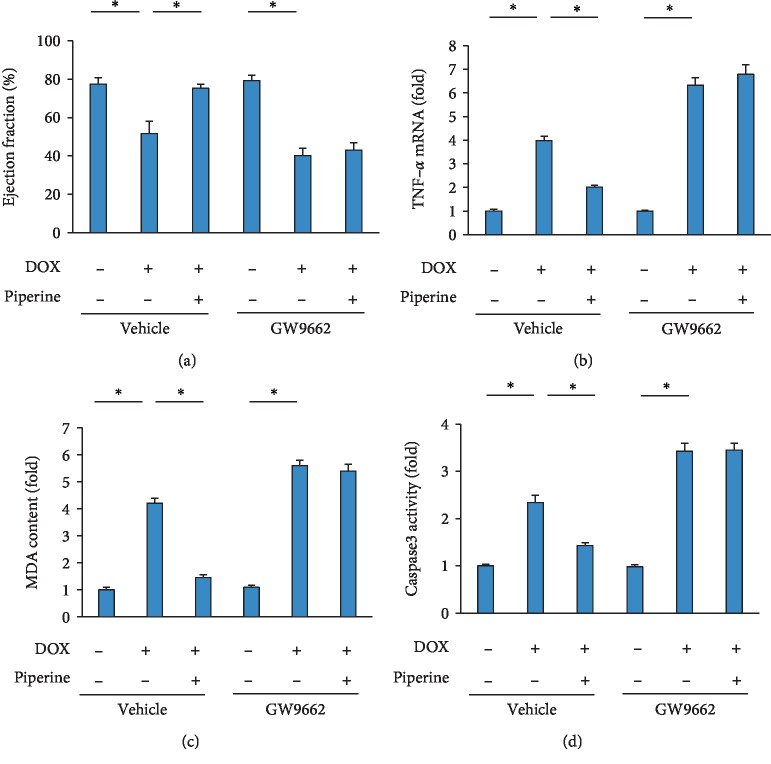
GW9662 abolished effects of piperine treatment in mice. (a) Ejection fraction (*n* = 8). (b) TNF-*α* mRNA level (*n* = 8). (c) MDA level (*n* = 8). (d) Caspase3 activity (*n* = 8). Data are expressed as mean ± SD. ^∗^*P* < 0.05. Data were analyzed sing one-way ANOVA, followed by Tukey post hoc analysis.

To further confirm the role of PPAR-*γ*, mice were treated with a PPAR-*γ* inhibitor (GW9662, 0.35 mg/kg per day in drinking water). The data in our study demonstrated that there was no difference in EF, TNF-*α*, MDA content and caspase3 activity between DOX+vehicle+GW9662 and DOX+piperine+GW9662 group (Figures 7(a)–7(d)).

## 4. Discussion

Our central finding is that piperine protected against DOX-induced cardiac injury and improved cardiac function in mice. Piperine also attenuated myocardial oxidative stress, inflammation and apoptosis in DOX-treated mice. We also found that piperine activated PPAR-*γ* receptors and PPAR-*γ* receptors inhibition could offset piperine-mediated protection in mice.

Accumulating evidence demonstrated that increased in oxidative stress and lipid peroxidation, along with reductions of antioxidants played key roles in the process of DOX-induced injury [21]. The heart samples are vulnerable to oxidative damage induced by DOX for the reason that there are a lot of mitochondrial content and relatively low levels of antioxidant enzymes [22]. Acute DOX injection significantly increased the levels of reactive oxygen species (ROS) and MDA level [23]. DOX also suppressed a variety of antioxidants, which further promoted oxidative stress in response to DOX treatment [24]. Here, we found that piperine markedly reduced MDA and 3-NT content, and improved SOD activity. We also found that piperine restored the level of Nrf2 in DOX-treated mice, and this finding was consistent with a previous study [25]. Accumulation of ROS in the hearts upregulated cardiac TNF-*α* accumulation, thus promoting the pathogenesis of DOX-related cardiac injury. As expected, we also found that piperine decreased myocardial inflammatory factors and nuclear NF-κB accumulation in mice, which was in line with a previous study that piperine inhibited lipopolysaccharide-induced inflammatory responses [12].

Death is the ultimate outcome of injured cells, and cell death is a direct cause of DOX-induced cardiac dysfunction. Accumulating evidence indicated that DOX caused apoptotic cell death among both endothelial cells and cardiomyocytes [5, 26]. In our study, we also found that piperine attenuated DOX-induced cardiac apoptosis in mice and improved cell viability in vitro. The inhibition of cell loss by piperine, at least partly, contributed to the protection of piperine against DOX-related injury.

It has been reported that activation of PPAR-*γ* could ameliorate palmitate-induced apoptosis in skeletal muscle cells [27]. DOX treatment significantly decreased the level of PPAR-*γ* [28]. These findings suggested that a PPAR-*γ* agonist might protect the mice from DOX-related cardiac injury. Consistent with our previous study [14], we found that piperine transactivated PPAR-*γ* and increased PPAR-*γ* mRNA in DOX-treated cells. Moreover, piperine could reduce DOX-related cardiac injury, as reflected by decreased cTnI and NT-proBNP, and improved cardiac function in mice. Moreover, these protective effects were blocked by GW9662 pretreatment, which is an irreversible antagonist of PPAR-*γ*, implying piperine exerted its cardioprotection via activating PPAR-*γ*.

It has been accepted that rosiglitazone and pioglitazone are agonists of PPAR-*γ*. However, their therapeutic use is limited by the potential cardiovascular risks including weight gain, which was largely caused by the high affinity of thiazolidinedione for PPAR-*γ* and the resultant PPAR-*γ* overactivation [29, 30]. In our previous study, we found the ability that piperine activated PPAR-*γ* pathway was less effective than pioglitazone [14], suggesting that that piperine partially activate PPAR-*γ*. Moreover, piperine did not affect body weight in mice without DOX injection, though piperine increased body weight in DOX-treated mice. In addition, it has been reported that piperine suppressed the growth of human melanoma cells in vivo and in vitro [31], implying that piperine might not compromise therapeutic DOX levels or promote tumor growth.

In conclusion, our study demonstrated that piperine protected against DOX-induced by cardiac injury via activating PPAR-*γ* pathway in mice. Our study provided experimental evidence for the clinical use of piperine in the treatment of DOX-related cardiac injury.

## Data Availability

The data in our study are available from the corresponding author upon reasonable request.

## References

[1] Vejpongsa P., Yeh E. T. (2014). Prevention of anthracycline-induced cardiotoxicity: challenges and opportunities. *Journal of the American College of Cardiology*.

[2] Carvalho C., Santos R. X., Cardoso S. (2009). Doxorubicin: the good, the bad and the ugly effect. *Current Medicinal Chemistry*.

[3] Ichikawa Y., Ghanefar M., Bayeva M. (2014). Cardiotoxicity of doxorubicin is mediated through mitochondrial iron accumulation. *Journal of Clinical Investigation*.

[4] Fan G. C., Zhou X., Wang X. (2008). Heat shock protein 20 interacting with phosphorylated Akt reduces doxorubicin-triggered oxidative stress and cardiotoxicity. *Circulation Research*.

[5] Yuan Y. P., Ma Z. G., Zhang X. (2018). CTRP3 protected against doxorubicin-induced cardiac dysfunction, inflammation and cell death via activation of Sirt1. *Journal of Molecular and Cellular Cardiology*.

[6] Matsuura N., Asano C., Nagasawa K. (2015). Effects of pioglitazone on cardiac and adipose tissue pathology in rats with metabolic syndrome. *International Journal of Cardiology*.

[7] Peng S., Xu J., Ruan W., Li S., Xiao F. (2017). PPAR-*?* activation prevents septic cardiac dysfunction via inhibition of apoptosis and necroptosis. *Oxidative Medicine and Cellular Longevity*.

[8] Czubryt M. P., McAnally J., Fishman G. I., Olson E. N. (2003). Regulation of peroxisome proliferator-activated receptor gamma coactivator 1 alpha (PGC-1 alpha ) and mitochondrial function by MEF2 and HDAC5. *Proceedings of the National Academy of Sciences*.

[9] Pakravan G., Foroughmand A. M., Peymani M. (2018). Downregulation of miR-130a, antagonized doxorubicin-induced cardiotoxicity via increasing the PPAR-*?*expression in mESCs-derived cardiac cells. *Cell Death & Disease*.

[10] Srinivasan K. (2007). Black pepper and its pungent principle-piperine: a review of diverse physiological effects. *Critical Reviews in Food Science and Nutrition*.

[11] Meghwal M., Goswami T. K. (2013). Piper nigrum and piperine: an update. *Phytotherapy Research*.

[12] Bae G. S., Kim M. S., Jung W. S. (2010). Inhibition of lipopolysaccharide-induced inflammatory responses by piperine. *European Journal of Pharmacology*.

[13] Liang Y. D., Bai W. J., Li C. G. (2016). Piperine suppresses pyroptosis and interleukin-1beta release upon ATP triggering and bacterial infection. *Frontiers in Pharmacology*.

[14] Ma Z. G., Yuan Y. P., Zhang X., Xu S. C., Wang S. S., Tang Q. Z. (2017). Piperine attenuates pathological cardiac fibrosis via PPAR-*?*/AKT pathways. *EBioMedicne*.

[15] Min L. J., Mogi M., Shudou M. (2012). Peroxisome proliferator-activated receptor-gamma activation with angiotensin II type 1 receptor blockade is pivotal for the prevention of blood-brain barrier impairment and cognitive decline in type 2 diabetic mice. *Hypertension*.

[16] Ma Z. G., Yuan Y. P., Xu S. C. (2017). CTRP3 attenuates cardiac dysfunction, inflammation, oxidative stress and cell death in diabetic cardiomyopathy in rats. *Diabetologia*.

[17] Ma Z. G., Dai J., Yuan Y. P. (2018). T-bet deficiency attenuates cardiac remodelling in rats. *Basic Research in Cardiology*.

[18] Ma Z. G., Yuan Y. P., Zhang X. (2019). C1q-tumour necrosis factor-related protein-3 exacerbates cardiac hypertrophy in mice. *Cardiovascular Research*.

[19] Ma Z. G., Zhang X., Yuan Y. P. (2018). A77 1726 (leflunomide) blocks and reverses cardiac hypertrophy and fibrosis in mice. *Clinical Science*.

[20] Konishi M., Haraguchi G., Ohigashi H. (2011). Adiponectin protects against doxorubicin-induced cardiomyopathy by anti-apoptotic effects through AMPK up-regulation. *Cardiovascular Research*.

[21] Benchekroun M. N., Pourquier P., Schott B., Robert J. (1993). Doxorubicin-induced lipid peroxidation and glutathione peroxidase activity in tumor cell lines selected for resistance to doxorubicin. *European Journal of Biochemistry*.

[22] Doroshow J. H., Locker G. Y., Myers C. E. (1980). Enzymatic defenses of the mouse heart against reactive oxygen metabolites: alterations produced by doxorubicin. *Journal of Clinical Investigation*.

[23] Takemura G., Fujiwara H. (2007). Doxorubicin-induced cardiomyopathy from the cardiotoxic mechanisms to management. *Progress in Cardiovascular Diseases*.

[24] Doroshow J. H., Locker G. Y., Baldinger J., Myers C. E. (1979). The effect of doxorubicin on hepatic and cardiac glutathione. *Research Communications in Chemical Pathology and Pharmacology*.

[25] Wang-Sheng C., Jie A., Jian-Jun L., Lan H., Zeng-Bao X., Chang-Qing L. (2017). Piperine attenuates lipopolysaccharide (LPS)-induced inflammatory responses in BV2 microglia. *International Immunopharmacology*.

[26] Kotamraju S., Konorev E. A., Joseph J., Kalyanaraman B. (2000). Doxorubicin-induced apoptosis in endothelial cells and cardiomyocytes is ameliorated by nitrone spin traps and ebselen. Role of reactive oxygen and nitrogen species. *Journal of Biological Chemistry*.

[27] Meshkani R., Sadeghi A., Taheripak G., Zarghooni M., Gerayesh-Nejad S., Bakhtiyari S. (2014). Rosiglitazone, a PPAR-*?* against, ameliorates palmitate-induced insulin resistance and apoptosis in skeletal muscle cells. *Cell Biochemistry and Function*.

[28] Arunachalam S., Tirupathi P. P., Achiraman S. (2013). Doxorubicin treatment inhibits PPAR-*?* and may induce lipotoxicity by mimicking a type 2 diabetes-like condition in rodent models. *FEBS Letters*.

[29] Larsen P. J., Lykkegaard K., Larsen L. K. (2008). Dissociation of antihyperglycaemic and adverse effects of partial perioxisome proliferator-activated receptor (PPAR-*?*) agonist balaglitazone. *European Journal of Pharmacology*.

[30] Nesto R. W., Bell D., Bonow R. O. (2004). Thiazolidinedione use, fluid retention, and congestive heart failure: a consensus statement from the american heart association and american diabetes association. *Diabetes Care*.

[31] Yoo E. S., Choo G. S., Kim S. H. (2019). Antitumor and apoptosis-inducing effects of piperine on human melanoma cells. *Anticancer Research*.

